# Epidemiological, behavioural, and clinical factors associated with antimicrobial-resistant gonorrhoea: a review

**DOI:** 10.12688/f1000research.13600.1

**Published:** 2018-03-27

**Authors:** Million Abraha, Dianne Egli-Gany, Nicola Low

**Affiliations:** 1 Institute of Social and Preventive Medicine, University of Bern, Finkenhubelweg 11, 3012 Bern, Switzerland

**Keywords:** Gonorrhoea, Neisseria gonorrhoeae, antimicrobial resistance, risk factors, epidemiology

## Abstract

Antimicrobial-resistant *Neisseria gonorrhoeae* is a global public health problem in the 21st century. *N. gonorrhoeae* has developed resistance to all classes of antibiotics used for empirical treatment, and clinical treatment failure caused by extensively resistant strains has been reported. Identifying specific factors associated with an increased risk of antimicrobial-resistant *N. gonorrhoeae* might help to develop strategies to improve antimicrobial stewardship. In this review, we describe the findings of 24 studies, published between 1989 and 2017, that examined epidemiological, behavioural, and clinical factors and their associations with a range of antimicrobial agents used to treat gonorrhoea. Antimicrobial-resistant *N. gonorrhoeae *is more common in older than younger adults and in men who have sex with men compared with heterosexual men and women. Antimicrobial-resistant *N. gonorrhoeae *is less common in some black minority and Aboriginal ethnic groups than in the majority white population in high-income countries. The factors associated with antimicrobial-resistant gonorrhoea are not necessarily those associated with a higher risk of gonorrhoea.

## Introduction

Antimicrobial-resistant *Neisseria gonorrhoeae* (AMR-NG) is a global public health challenge ^1^. The World Health Organization (WHO) estimates that, in 2012, more than 78 million new infections with gonorrhoea occurred worldwide ^2^. Of these, more than 90% were in low- and middle-income countries. In high-income countries, including England ^3^, the USA ^4^ and Australia ^5^, *N. gonorrhoeae* is the second most commonly reported bacterial sexually transmitted infection (STI). *N. gonorrhoeae* primarily infects the mucosal epithelium, causing urethritis in men, cervicitis in women, and rectal and pharyngeal infection in men who have sex with men (MSM) and women ^6^. Untreated infection that spreads to the upper genital tract can cause epididymo-orchitis and pelvic inflammatory disease, ectopic pregnancy, and tubal infertility ^6^. Infection in pregnancy is associated with preterm birth and low birthweight and can cause neonatal conjunctivitis if transmitted during delivery. Rarely, *N. gonorrhoeae* can ** spread systemically, causing arthritis, endocarditis, and septicaemia. The inflammatory response to *N. gonorrhoeae* in the genital tract increases the infectivity of HIV. All of these complications will become more frequent if antimicrobial resistance renders gonorrhoea untreatable. Gonorrhoea shares some epidemiological characteristics with other bacterial STIs ^7^. It is associated with higher numbers of sex partners ^8^ (which are more common in MSM than in heterosexual adults ^9, 10^), younger age ^3^, and lower socioeconomic position ^11^, and, in high-income countries, it is associated with being a member of some black and ethnic minority groups ^11^.

*N. gonorrhoeae* is a bacterium that has extensive capacity for genetic mutation or plasmid exchange of resistant genes throughout its life cycle ^1^. This remarkable biological characteristic has helped the bacteria to survive and evolve or acquire resistance to many different classes of antibiotics over the years ^1^. Unemo and Shafer have reviewed antimicrobial treatments for gonorrhoea and the emergence of resistance comprehensively up to 2014 ^1^. Penicillin was first used to treat gonorrhoea in 1943. Initially, chromosomally mediated resistance emerged, so higher and higher doses were needed to cure gonorrhoea. In 1976, the first plasmid-mediated penicillinase-producing strains were reported from South East Asia and West Africa ^12, 13^. In the 1990s, quinolones, particularly the fluoroquinolone ciprofloxacin, replaced penicillin as the first-line treatment for gonorrhoea ^14^. Resistance was reported initially from countries in South East Asia and spread internationally by the early to mid-2000s. Third-generation, extended-spectrum cephalosporins (ESCs) (mostly oral cefixime and injectable ceftriaxone) have been recommended for first-line use since the early 2000s. Resistance to ESCs was reported first in Japan ^15^, and strains with high-level resistance to ESCs spread to Europe ^16– 19^. Currently, the WHO recommends dual therapy with ceftriaxone and azithromycin for the first-line treatment of gonorrhoea, and the intention is to ensure cure rates of greater than 95% of infections ^20^. Clinical treatment failure and high-level resistance to this regimen were reported in 2016 ^17^. Resistance has also emerged to other drugs, such as tetracyclines, spectinomycin, and azithromycin, that have not been used widely as first-line treatments.

Antimicrobial resistance hampers strategies to control and prevent gonorrhoea ^21^. Understanding factors that are associated with AMR-NG could help to identify groups at high risk of having resistant infections, provide more focused management, and assist antimicrobial stewardship. In this review, we describe the findings of studies that have examined associations between epidemiological, behavioural, and clinical factors and the presence of AMR-NG.

## Search strategy

We searched Medline (Ovid, Wolters Kluwer) from 1946 until August 2017 without language restrictions by using combinations of keywords for the organism, AMR, and associated factors: *Neisseria gonorrhoeae* or gonorrhoea, drug resistance, risk factors, sexual behaviour, health services, or epidemiology. We selected studies that compared epidemiological, behavioural, or clinical factors in people with or without AMR-NG. We recorded information about study characteristics, study population, antimicrobials, and findings from each study in an evidence table ( Appendix 1).

## Characteristics of included studies

Of 129 articles identified, 24 publications were included ^14, 22– 44^. Appendix 1 summarises the main characteristics of each study. All included studies used a cross-sectional (16 studies) or case-control (eight studies) study design. Nine studies were nested in surveillance systems for AMR-NG ^23, 25, 28, 30, 32, 37– 39, 44^, and 14 reported a multivariable analysis ^23– 25, 27, 28, 30, 31, 35, 36, 39, 40, 42– 44^. The evidence that we found about factors associated with AMR-NG comes mainly from regions and countries that do not have the highest incidence of gonorrhoea ( Table 1). Of 24 included studies, 19 came from Europe ^23, 25, 27– 29, 35, 37, 40, 42– 44^ and North America ^14, 22, 26, 30, 33, 38, 39, 41^, although the WHO European Region and the whole WHO Region of the Americas account for only 20% of people with incident gonorrhoea worldwide ^2^. These regions include countries with the best-established surveillance systems for STIs in general and systematic surveillance systems for antimicrobial resistance, such as the Gonococcal Resistance to Antimicrobials Surveillance Programme (GRASP) in England and Wales ^23^, the US Gonococcal Isolate Surveillance Program (GISP) ^30^, and the Australian Gonococcal Surveillance Programme (AGSP) ^32^. These systems can collect demographic and epidemiological data so that associations with AMR-NG can be assessed regularly. Our search did not find any studies about potential risk factors for AMR-NG from Africa, where the prevalence and incidence of gonorrhoea are high ^2^, or from Latin America and the Caribbean, South East Asia, or Eastern Mediterranean regions, where surveillance for STIs and AMR is also limited. Although AMR-NG strains with resistance to penicillin (penicillinase-producing), spectinomycin, fluoroquinolones, and ESCs were first reported from countries in the Western Pacific region, such as Japan, South Korea, and the Philippines ^45^, we found only three studies in the region that examined factors associated with AMR-NG: two in China ^24, 36^ and one in the Philippines ^31^.

**Table 1.** Estimated numbers of new gonorrhoea cases, countries reporting *Neisseria gonorrhoeae* resistance, and number of studies about risk factors by WHO region

WHO region ^a^(number of countries in the region)	Number of new cases of gonorrhoea, 2012 ^b^	Countries reporting resistance for at least one year from 2009 to 2014 ^c^			Number of publications in review
		Ciprofloxacin	Azithromycin	ESC	
African region (46 countries)	11,440	6/8	3/3	1/9	0
Region of the Americas (35 countries)	10,974	14/16	2/7	0/16	
North America					8
Latin America and Caribbean					0
Eastern Mediterranean region (21 countries)	4,526	0/1	0/1	0/3	0
European region (53 countries)	4,686	23/26	21/26	15/27	11
South East Asia region (11 countries)	11,407	2/6	1/6	4/6	0
Western Pacific region (27 countries)	35,247	7/15	2/15	6/16	
Australia					2
Rest of Western Pacific					3

^a^Region of the Americas divided into countries of North America and Latin America and the Caribbean; all studies in the review were in North America. The Western Pacific region subdivided into Australia and all other countries in the region; two out of five studies were from Australia.

^b^Estimates from the World Health Organization (WHO) ^2^. The point estimate for each region is given. Numbers of cases for the subdivided regions are not known.

^c^From WHO global gonococcal antimicrobial surveillance programme (GASP) ^21^. Numbers are the number of countries reporting resistance of at least 5%/total number countries reporting to GASP. ESC, extended-spectrum cephalosporin.

**Figure 1.**
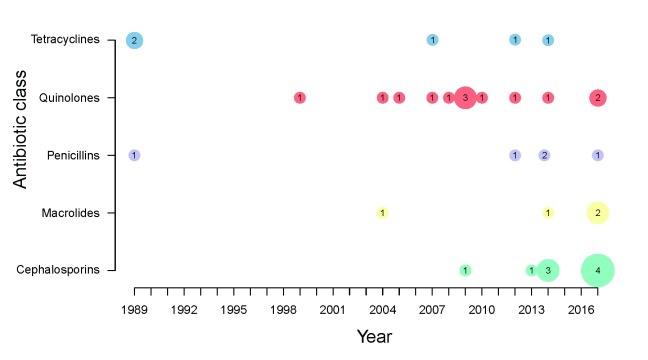
Number of studies found about factors associated with antimicrobial-resistant *Neisseria gonorrhoeae* from 1989 to 2017, according to class of antimicrobial.

Figure 1 shows the distribution of studies that have examined potential risk factors for AMR-NG over time, according to antibiotic class. Broadly speaking, these follow the periods in which each antimicrobial class was a recommended treatment. The first studies, published in 1989, examined risk factors for penicillin resistance and for tetracycline, which was beginning to be used to treat chlamydia infections and non-specific genital infections ^22, 33^. The next, and largest, group of studies focused on the identification of factors potentially associated with resistance to fluoroquinolones ^14, 26, 29, 31, 39– 41^, followed by macrolides ^25, 27, 37, 38^ and ESCs ^23– 25, 27, 28, 35– 37, 42^.

## Factors potentially associated with antimicrobial-resistant *Neisseria gonorrhoeae*

We describe epidemiological, behavioural, and clinical factors that have been examined in association with AMR-NG (summarised in Appendix 2). We describe as ‘risk factors’ factors associated with an increased risk or odds of AMR-NG, based on the effect size and its 95% confidence intervals (CIs), where available. Our overall interpretation takes into account the size of the study and the type of statistical analysis. Where findings between studies are inconsistent, we give more emphasis to findings from larger studies with multivariable analyses that control for important potential confounding factors. For most factors examined, there were too few studies to determine whether associations differ for different antimicrobials. In observational studies, confounding of observed associations by measured or unmeasured factors is likely.

### Epidemiological factors

***Age.*** Younger age is a risk factor for gonorrhoea; the peak age groups for diagnosis of gonorrhoea are 20–24 years in both women and men in the USA ^4^ and 20–24 in women and 25–35 in men in England ^3^. Amongst MSM, the peak age at infection is somewhat older ^3^.

In contrast, AMR-NG was more common in adults who were 25 years or older than in younger people in most studies that examined age as a risk factor for resistance to tetracyclines, fluoroquinolones, and ESCs ( Appendix 2). This finding might have resulted from the inclusion of large numbers of MSM; in two large studies, age was no longer associated with decreased susceptibility to ESCs in multivariable analyses adjusted for the composition of the study population ^23, 42^. In several studies, however, older age remained associated with AMR-NG in multivariable analyses, including ciprofloxacin resistance in women in the Netherlands ^44^; reduced susceptibility to ceftriaxone in heterosexual men and women, but not MSM, in England and Wales ^28^; ciprofloxacin resistance in Spain ^35^; ciprofloxacin and cefixime but not azithromycin resistance in a European Union surveillance network ^25^; and probable resistance to ceftriaxone but not to penicillin or tetracycline in China ^24^. Studies that found no association or an association with younger age were small or methodologically flawed ^36^.

***Sex.*** Gonorrhoea surveillance reports show higher numbers of reported cases of gonorrhoea in men than in women ^3– 5^, even after the high proportion of infections diagnosed in MSM was taken into account ^3, 4^. The higher frequency of symptomatic infections in men than in women results in higher levels of attendance at healthcare settings ^6^. We found 11 studies that compared AMR-NG between heterosexual men and women ^14, 23– 25, 27– 29, 33, 35, 36, 41^. Three publications from two studies with multivariable analyses found AMR-NG more commonly in heterosexual men than in women ^23, 24, 28^. Heterosexual men had about twice the odds of NG with reduced susceptibility to ceftriaxone than did women in China ^24^ and in England and Wales ^23, 28^ and of chromosomal resistance to tetracycline in China (adjusted odds ratio [OR] 2.73, 95% CI 1.06–7.05) ^24^.

***Same-sex sexual partnerships in men.*** Gonorrhoea is more commonly reported in MSM than in men who have sex with women only or in women ^46^, and rates of reported gonorrhoea are increasing more rapidly in MSM than in men who have sex with women only or in women ^46, 47^. Most studies that have examined this factor ^14, 23, 35, 44^ also found that AMR-NG was more common in MSM than in men who have sex with women only, including most studies with multivariable analyses ^23, 35, 42, 44^. AMR-NG was more commonly found in MSM compared with men who have sex with only women in the Netherlands for cefotaxime (age-adjusted OR 2.9, 95% CI 1.4–5.8) ^42^ and ciprofloxacin (adjusted OR 2.0, 95% CI 1.5–2.6) ^44^, in England and Wales for cefixime (adjusted OR 5.47, 95% CI 3.99–7.48) ^23^, and in some but not all counties in California (USA) for ciprofloxacin ^39^. It has been hypothesised that, in the USA, the emergence of resistance to ciprofloxacin started in MSM and spread to men who have sex with women only ^30^. In the European Gonococcal Antimicrobial Resistance Programme (Euro-GASP), covering more than 20 countries, AMR-NG was reported to be more common among heterosexual men than among MSM ^25, 37^. The reasons for the discrepant findings are not clear because the data were aggregated across all countries and were missing from nearly half of the records ^25^.

AMR-NG might also be common in MSM because the pharynx is thought to be a reservoir for strains that have acquired genes that confer resistance to ESCs in commensal Neisseria species (see ‘Anatomical site of infection’ subsection of ‘Clinical factors’ section) ^1^. MSM can have gonococcal infection in the pharynx and rectum, resulting from oral and anal sexual intercourse, as well as the urethra ^6, 48^. Pharyngeal and rectal gonorrhoea are usually asymptomatic and can remain untreated if these anatomical sites are not sampled ^49^. Anatomical site of infection is considered as a risk factor below.

***Racial or ethnic group.*** Surveillance reports show that rates of gonorrhoea diagnoses are several times higher in some minorities, such as African American, black Caribbean, and indigenous Aboriginal ethnic groups, than in the majority white population in countries such as the USA ^4^, the UK ^3^, the Netherlands ^50^, Canada ^26^, and Australia ^5^. We found eight studies that examined racial or ethnic group as a risk factor ^14, 23, 26– 28, 32, 39, 44^. AMR-NG was not more common in black and Aboriginal ethnic groups. Ciprofloxacin resistance was less common in people from black, Hispanic, and other ethnic groups than in whites in a multivariable analysis in the USA ^39^ and less common in people from Aboriginal groups in Canada in a univariable analysis ^26^. Decreased susceptibility was less common in people from ethnic groups in multivariable analyses in England and Wales ^23, 28^. In Australia, surveillance data from the Northern Territories and Western Australia showed a much lower proportion of penicillinase-producing NG isolates in remote areas (2%), in which the population is almost entirely Aboriginal, than in urban areas (14–19%), where the population is mixed ^32^. However, in the USA, ciprofloxacin was slightly more common in people from Asian and Pacific Island ethnic groups than in whites ^14, 39^. Assortative sexual mixing patterns, in which people are more likely to have partners from their own than from other ethnic groups ^51^, are likely to contribute to differential rates of both gonorrhoea infection and maybe also AMR-NG.

***Socioeconomic position.*** Whilst higher rates of reported gonorrhoea are strongly associated with lower socioeconomic position, possibly as a marker of poor education and awareness of STIs ^11^ and limited access to healthcare, we found only one study that had examined the association with AMR-NG. In one study in China, higher income levels were associated with lower levels of plasmid-mediated tetracycline resistance (adjusted OR 0.34, 95% CI 0.14–0.18) but not ceftriaxone or penicillin resistance ^24^.

### Behavioural factors

***Multiple sex partners.*** Gonorrhoea has a short duration of infectiousness, and its persistence in a population relies on transmission in groups with high rates of sexual partner change ^8^. The probability of acquiring AMR-NG, however, is not necessarily associated with higher numbers of sexual partners when other factors are taken into consideration. In several studies, a higher number of sexual partners was associated with AMR-NG in univariable analysis ^22– 24, 27, 28, 35, 38, 40^. In studies that conducted multivariable analyses ^23, 27, 35^, only one study, in the Netherlands, found that the association persisted, with an attenuated OR ^27^.

***Sex with partners abroad.*** Travel abroad has been reported in some studies as a risk factor for STIs ^52, 53^, presumably because people take more risks when on holiday, such as having unprotected sex with casual partners ^54^. Since AMR-NG often arises first in countries in South East Asia and the Western Pacific, travellers, including sex tourists, who have unprotected sex in these regions are assumed to import AMR-NG into their home countries ^45, 55^. We found 10 studies that investigated travel or sexual contact abroad as a risk factor for AMR-NG ^14, 23, 27– 30, 35, 38, 39, 43^, and four of them examined fluoroquinolone resistance in the late 1990s and early 2000s ^14, 30, 39, 43^. Ciprofloxacin resistance was more common in those reporting travel abroad or sex with a partner who had travelled abroad in univariable analyses from Hawaii ^14^ and California ^39^ but not in multivariable analysis ^39^. A national study in the USA found higher levels of fluoroquinolone resistance in heterosexual men with a history of travel but found lower levels in MSM ^30^. Another study found an association, in multivariable analysis, with sexual contact outside Switzerland ^43^. The variables in these studies do not specify exposures in particular places and might underestimate associations. Supportive evidence about the international spread of AMR-NG comes from gene sequencing studies of some highly resistant *N. gonorrhoeae* clonal strains ^18^. More detailed studies on people with gonorrhoea and their sexual networks with detailed phenotypic and genotypic characterisation would contribute to the identification of the origin and spread of resistance.

***Exchanging sex for money.*** Commercial sex workers and their clients in some countries are at high risk of acquiring STIs, including gonorrhoea ^1^. We included seven studies that considered commercial sex and AMR-NG ^14, 22, 31, 33, 38, 39, 44^. One of these studies, conducted among female commercial sex workers in the Philippines from 1996 to 1997, found that, in multivariable analysis, high-level resistance to ciprofloxacin was associated with living in the capital, Manila, and having recently started sex work ^31^. One study in the Netherlands found that, in multivariable analysis, female sex workers had a much higher risk of ciprofloxacin-resistant gonorrhoea than did other women (adjusted OR 25.0, 95% CI 7.7–78.2) ^44^. Studies in the USA did not distinguish clearly between female or male sex workers or clients ^14, 22, 38, 39^; exposure to commercial sex work was associated with AMR-NG in univariable analysis in only two studies ^22, 38^.

***Alcohol and drug use.*** Only four of the included studies ^22, 24, 38, 39^ looked at these factors. One study in China found that alcohol use was associated with tetracycline resistance (adjusted OR 1.69, 95% CI 1.08–2.64) ^24^ in multivariable analysis. In the USA, one study found that having had a sex partner who received drugs or money for sex was associated with azithromycin resistance (crude OR 34.0, 95% CI 2.3–1651) ^38^, but another study found a much weaker association with ciprofloxacin resistance in univariable analysis and no association in multivariable analysis ^39^. These factors warrant more detailed investigation.

### Clinical factors

***Anatomical site of infection.*** MSM and commercial sex workers can harbour *N. gonorrhoeae* in the pharynx ^1, 6^. We found three studies ^25, 27, 28^ that considered anatomical site of infection. All three studies conducted multivariable analysis. In the Netherlands, ceftriaxone resistance was more common in the pharynx than in the urethra amongst MSM (adjusted OR 2.52, 95% CI 1.64–3.89) but not heterosexual women and men ^27^, and in England and Wales, a slight decrease in susceptibility to ceftriaxone was more common in the pharynx in heterosexual women and men (adjusted OR 1.84, 95% CI 1.44–2.34) but not MSM ^23^. In Euro-GASP, isolates from the pharynx were not more likely than genital isolates to show AMR-NG, but cefixime and ciprofloxacin resistance were reported to be less common in anorectal than in genital isolates ^25^.

***Co-infection with HIV and other sexually transmitted infections.*** People infected with NG are at higher risk of acquiring HIV infection ^56^. Being co-infected with HIV was associated with resistance to ESCs or ciprofloxacin in univariable but not multivariable analysis in three studies in the Netherlands and in England and Wales ^23, 42, 44^. In another study in the Netherlands, MSM with HIV infection were less likely than HIV-negative MSM to have azithromycin resistance (adjusted OR 0.72, 95% CI 0.54–0.96) in multivariable analysis ^27^. Co-infection with *Chlamydia trachomatis* is also common in people with gonorrhoea. In studies conducted by GRASP in England and Wales ^23, 28^, people who were not co-infected with chlamydia were more likely to have AMR-NG in multivariable analyses. There is no definitive explanation for this finding.

***Recent antibiotic use.*** Antimicrobial use exerts selection pressure for the emergence of resistance ^1^. Current or recent antimicrobial use was examined in five studies in the USA, but findings were inconsistent ^14, 22, 33, 38, 39^. Ciprofloxacin resistance was found more commonly in female sex workers in the Philippines who were taking antimicrobials in univariable but not multivariable analysis ^31^. Studies that did not find associations with past antimicrobial use might have asked questions that were not specific enough about particular antimicrobials.

### Other risk factors

Additional factors—such as gonorrhoea or STI history, lifetime sex partners, partnership type, more than one infected site, and year of isolation—that were reported in small numbers of studies are listed in Appendix 2 but are not described in detail here.

## Discussion

In this review, AMR-NG was more common in older than in younger adults, in heterosexual men than in women, in MSM compared with men who have sex with women only, and possibly in people with poor socioeconomic position. People from some black ethnic groups in the USA and Europe and Aboriginal ethnic groups living in Canada and Australia are less likely to have AMR-NG than the white majority population. Very few studies about risk factors for AMR-NG have been done in countries in sub-Saharan Africa, Latin America, or some parts of South East Asia and the Western Pacific where gonorrhoea is most common.

The main strength of this review is that we searched for studies worldwide, irrespective of the language and the year of publication, and we extracted the same information from all studies. The main limitation of the review is that it was not entirely systematic. Our search of Medline might have missed studies, particularly from low- and middle-income countries, non-English language journals, and grey literature. Therefore, the findings of the review are most applicable to factors associated with antimicrobial-resistant gonorrhoea in high-income countries in Europe, North America, and Australia. We did not follow a protocol, and, although we selected factors of interest in advance, we did not report all study findings comprehensively. Nevertheless, our interpretation took into account studies that found no association with a potential risk factor and we distinguished between associations found only in univariable analyses and those found consistently in multivariable analyses that control for potential confounding factors.

This review shows that some risk factors for AMR-NG are not necessarily those associated with a higher risk of gonorrhoea infection itself ( Appendix 3). Of note, whilst the risk of gonorrhoea in heterosexual adults is highest amongst younger people with high numbers of sexual partners, AMR-NG appears to be more common in older adults and, after other factors were controlled for, high numbers of sexual partners were not consistently associated with AMR-NG. AMR-NG was also less likely amongst people from black minority and Aboriginal ethnic groups living in countries where the majority of the population is from white ethnic groups. These findings appeared to be consistent across several different antibiotic classes. We cannot provide definitive explanations for these findings, but they could offer some empirical support for the results of a mathematical modelling study, which found that a high treatment rate, rather than the rate of partner change, predicts the spread of AMR-NG ^57^. Higher prevalence of AMR-NG in MSM could result from a combination of factors, including a high risk of gonorrhoea infection at older ages than in heterosexuals ^3^, frequent oral sex resulting in pharyngeal infections ^6^, and high attendance rates at sexual health clinics ^58^.

We did not find that recent travel abroad, commonly reported as a risk factor for AMR-NG, was consistently associated with resistance. Because a history of recent travel, as asked about in the US GISP, is too non-specific, some studies might not have found an association. In addition, associations might differ over time and be found when resistance to a particular class of antimicrobials, or a specific gonococcal clone, starts to spread but might not be found at a later time point. Evidence from gene sequencing studies with supportive evidence from epidemiological studies strongly suggests that antimicrobial-resistant gonococcal strains appear to emerge in parts of South East Asia and are spread by international travellers ^1^. Researchers have found more consistent evidence of the role of travel for other organisms. A systematic review of cohort studies showed high levels of acquisition of multidrug-resistant *Enterobacteriaceae* in travellers returning from countries in southern Asia ^59^.

## Conclusions and recommendations for future research

This review found a limited number of studies that investigated factors associated with AMR-NG and few studies from low- and middle-income countries where both gonorrhoea and antimicrobial resistance are most common. For this reason, we could not provide a comprehensive global picture of factors that increase the risk of AMR-NG. The factors associated with antimicrobial-resistant gonorrhoea are not necessarily those associated with a higher risk of gonorrhoea. Future research studies should investigate in more detail the apparent associations with increased risk of AMR-NG in older age groups and amongst travellers and with decreased risk of AMR-NG in black and Aboriginal groups living in high-income countries. Improvements in surveillance systems for antimicrobial resistance, including enhanced surveillance that collects information about key factors such as age, same sex partnerships, travel-associated sexual partnerships, or sentinel surveillance in specific groups, might allow earlier identification of emerging resistance and of risk factors that could allow more intensive follow-up and prevention interventions in groups at high risk of AMR-NG ^21^. Better knowledge about modifiable risk factors for AMR-NG could help to mitigate the spread of resistance to ESCs, the last recommended empirical treatment for gonorrhoea.

## Supplementary material

Supplementary file 1. List of articles included in review, by year of publication.

Supplementary file 2. Risk factors examined and associations with AMR-NG, as reported by authors of included studies.

Supplementary file 3. Comparison between risk factors for gonorrhoea and factors associated with AMR-NG in this review.

## References

[1] Unemo M Shafer WM: Antimicrobial resistance in *Neisseria gonorrhoeae* in the 21st century: past, evolution, and future. *Clin Microbiol Rev.* 2014;27(3):587–613. 10.1128/CMR.00010-14 24982323 PMC4135894

[2] Newman L Rowley J Vander Hoorn S: Global Estimates of the Prevalence and Incidence of Four Curable Sexually Transmitted Infections in 2012 Based on Systematic Review and Global Reporting. *PLoS One.* 2015;10(12):e0143304. 10.1371/journal.pone.0143304 26646541 PMC4672879

[3] Public Health England: Sexually transmitted infections (STIs): annual data tables - GOV.UK. Sexually transmitted infections (STIs): annual data tables 2017. Accessed November 29, 2017. Reference Source

[4] Centers for Disease Control and Prevention: Gonorrhea - 2016 STD Surveillance Report.2016 Accessed November 29, 2017. Reference Source

[5] HIV, Viral Hepatitis and Sexually Transmissible Infections in Australia: Annual Surveillance Report 2017. Sydney: UNSW Sydney;2017 Accessed November 29, 2017. Reference Source

[6] Hook EW III Handsfield HH: Gonococcal Infections in the Adult. In Holmes KK, Sparling F, Stamm WE, *et al.*, eds. *Sexually Transmitted Diseases* 4th ed. New York, N.Y.: McGraw-Hill;2008;627–642.

[7] Bjekić M Vlajinac H Sipetić S: Risk factors for gonorrhoea: case-control study. *Genitourin Med.* 1997;73(6):518–21. 10.1136/sti.73.6.518 9582473 PMC1195937

[8] Van Duynhoven YT van de Laar MJ Schop WA: Different demographic and sexual correlates for chlamydial infection and gonorrhoea in Rotterdam. *Int J Epidemiol.* 1997;26(6):1373–85. 10.1093/ije/26.6.1373 9447420

[9] Kissinger P Sanusi A Bell DL Issues in Men’s Reproductive Health. *Sexually Transmitted Diseases (Second Edition)* Chapter 7.2013;165–188, Accessed October 24, 2017. 10.1016/B978-0-12-391059-2.00007-3

[10] Judson FN Penley KA Robinson ME: Comparative prevalence rates of sexually transmitted diseases in heterosexual and homosexual men. *Am J Epidemiol.* 1980;112(6):836–43. 10.1093/oxfordjournals.aje.a113056 6893897

[11] Harling G Subramanian S Bärnighausen T: Socioeconomic disparities in sexually transmitted infections among young adults in the United States: examining the interaction between income and race/ethnicity. *Sex Transm Dis.* 2013;40(7):575–81. 10.1097/OLQ.0b013e31829529cf 23965773 PMC3752095

[12] Percival A Rowlands J Corkill JE: Penicillinase-producing Gonococci in Liverpool. *Lancet.* 1976;2(8000):1379–82. 10.1016/S0140-6736(76)91919-X 63850

[13] Phillips I: Beta-lactamase-producing, penicillin-resistant gonococcus. *Lancet.* 1976;2(7987):656–7. 10.1016/S0140-6736(76)92466-1 60518

[14] Iverson CJ Wang SA Lee MV: Fluoroquinolone resistance among *Neisseria gonorrhoeae* isolates in Hawaii, 1990–2000: role of foreign importation and increasing endemic spread. *Sex Transm Dis.* 2004;31(12):702–8. 10.1097/01.olq.0000145846.45781.a4 15608583

[15] Ohnishi M Saika T Hoshina S: Ceftriaxone-resistant *Neisseria gonorrhoeae*, Japan. *Emerging Infect Dis.* 2011;17(1):148–9. 10.3201/eid1701.100397 21192886 PMC3204624

[16] Unemo M Golparian D Potočnik M: Treatment failure of pharyngeal gonorrhoea with internationally recommended first-line ceftriaxone verified in Slovenia, September 2011. *Euro Surveill.* 2012;17(25): pii: 20200. 22748003

[17] Fifer H Natarajan U Jones L: Failure of Dual Antimicrobial Therapy in Treatment of Gonorrhea. *N Engl J Med.* 2016;374(25):2504–6. 10.1056/NEJMc1512757 27332921

[18] Unemo M Golparian D Nicholas R: High-level cefixime- and ceftriaxone-resistant *Neisseria gonorrhoeae* in France: novel *penA* mosaic allele in a successful international clone causes treatment failure. *Antimicrob Agents Chemother.* 2012;56(3):1273–80. 10.1128/AAC.05760-11 22155830 PMC3294892

[19] Unemo M Golparian D Hestner A: Ceftriaxone treatment failure of pharyngeal gonorrhoea verified by international recommendations, Sweden, July 2010. *Euro Surveill.* 2011;16(6): pii: 19792. 21329645

[20] WHO: WHO guidelines for the treatment of *Neisseria gonorrhoeae* .2016 Accessed October 17, 2017. Reference Source 27512795

[21] Wi T Lahra MM Ndowa F: Antimicrobial resistance in *Neisseria gonorrhoeae*: Global surveillance and a call for international collaborative action. *PLoS Med.* 2017;14(7):e1002344. 10.1371/journal.pmed.1002344 28686231 PMC5501266

[22] Hook EW 3rd Brady WE Reichart CA: Determinants of emergence of antibiotic-resistant *Neisseria gonorrhoeae*. *J Infect Dis.* 1989;159(5):900–7. 10.1093/infdis/159.5.900 2496174

[23] Ison CA Town K Obi C: Decreased susceptibility to cephalosporins among gonococci: data from the Gonococcal Resistance to Antimicrobials Surveillance Programme (GRASP) in England and Wales, 2007–2011. *Lancet Infect Dis.* 2013;13(9):762–8. 10.1016/S1473-3099(13)70143-9 23764300

[24] Trecker MA Waldner C Jolly A: Behavioral and socioeconomic risk factors associated with probable resistance to ceftriaxone and resistance to penicillin and tetracycline in *Neisseria gonorrhoeae* in Shanghai. *PLoS One.* 2014;9(2):e89458. 10.1371/journal.pone.0089458 24586792 PMC3929748

[25] Cole MJ Spiteri G Town K: Risk factors for antimicrobial-resistant *Neisseria gonorrhoeae* in Europe. *Sex Transm Dis.* 2014;41(12):723–9. 10.1097/OLQ.0000000000000185 25581808

[26] Plitt S Boyington C Sutherland K: Antimicrobial resistance in gonorrhea: the influence of epidemiologic and laboratory surveillance data on treatment guidelines: Alberta, Canada 2001–2007. *Sex Transm Dis.* 2009;36(10):665–9. 10.1097/OLQ.0b013e3181aad9df 19704400

[27] Wind CM Schim van der Loeff MF van Dam AP: Trends in antimicrobial susceptibility for azithromycin and ceftriaxone in *Neisseria gonorrhoeae* isolates in Amsterdam, the Netherlands, between 2012 and 2015. *Euro Surveill.* 2017;22(1): pii: 30431. 10.2807/1560-7917.ES.2017.22.1.30431 28079519 PMC5388096

[28] Town K Obi C Quaye N: Drifting towards ceftriaxone treatment failure in gonorrhoea: risk factor analysis of data from the Gonococcal Resistance to Antimicrobials Surveillance Programme in England and Wales. *Sex Transm Infect.* 2017;93(1):39–45. 10.1136/sextrans-2016-052583 27382010

[29] Farhi D Hotz C Poupet H: Neisseria gonorrhoeae antibiotic resistance in Paris, 2005 to 2007: implications for treatment guidelines. *Acta Derm Venereol.* 2009;89(5):484–7. 10.2340/00015555-0704 19734973

[30] Goldstein E Kirkcaldy RD Reshef D: Factors related to increasing prevalence of resistance to ciprofloxacin and other antimicrobial drugs in *Neisseria gonorrhoeae*, United States. *Emerging Infect Dis.* 2012;18(8):1290–7. 10.3201/eid1808.111202 22840274 PMC3414012

[31] Klausner JD Aplasca MR Mesola VP: Correlates of gonococcal infection and of antimicrobial-resistant *Neisseria gonorrhoeae* among female sex workers, Republic of the Philippines, 1996–1997. *J Infect Dis.* 1999;179(3):729–33. 10.1086/314625 9952388

[32] Lahra MM Enriquez RP National Neisseria Network: Australian Gonococcal Surveillance Programme annual report, 2015. *Commun Dis Intell Q Rep.* 2017;41(1):E. 28385139 10.33321/cdi.2017.41.9

[33] Telzak EE Spitalny KC Faur YC: Risk factors for infection with plasmid-mediated high-level tetracycline resistant Neisseria gonorrhoeae. *Sex Transm Dis.* 1989;16(3):132–6. 10.1097/00007435-198907000-00003 2510326

[34] Speers DJ Fisk RE Goire N: Non-culture *Neisseria gonorrhoeae* molecular penicillinase production surveillance demonstrates the long-term success of empirical dual therapy and informs gonorrhoea management guidelines in a highly endemic setting. *J Antimicrob Chemother.* 2014;69(5):1243–7. 10.1093/jac/dkt501 24379305

[35] Fuertes de Vega I Baliu-Piqué C Bosch Mestres J: Risk factors for antimicrobial-resistant *Neisseria gonorrhoeae* and characteristics of patients infected with gonorrhea. *Enferm Infecc Microbiol Clin.* 2018;36(3):165–168. 10.1016/j.eimc.2016.11.012 28094065

[36] Zhu BY Yu RX Yin Y: Surveillance of antimicrobial susceptibilities of Neisseria gonorrhoeae in Nanning, China, 2000 to 2012. *Sex Transm Dis.* 2014;41(8):501–6. 10.1097/OLQ.0000000000000157 25013979

[37] Cole MJ Spiteri G Jacobsson S: Overall Low Extended-Spectrum Cephalosporin Resistance but high Azithromycin Resistance in *Neisseria gonorrhoeae* in 24 European Countries, 2015. *BMC Infect Dis.* 2017;17(1):617. 10.1186/s12879-017-2707-z 28893203 PMC5594611

[38] McLean CA Wang SA Hoff GL: The emergence of Neisseria gonorrhoeae with decreased susceptibility to Azithromycin in Kansas City, Missouri, 1999 to 2000. *Sex Transm Dis.* 2004;31(2):73–8. 10.1097/01.OLQ.0000109514.91508.FC 14743069

[39] Bauer HM Mark KE Samuel M: Prevalence of and associated risk factors for fluoroquinolone-resistant *Neisseria gonorrhoeae* in California, 2000–2003. *Clin Infect Dis.* 2005;41(6):795–803. 10.1086/432801 16107976

[40] Farhi D Gerhardt P Falissard B: Increasing rates of quinolone-resistant *Neisseria gonorrhoeae* in Paris, France. *J Eur Acad Dermatol Venereol.* 2007;21(6):818–21. 10.1111/j.1468-3083.2006.02054.x 17567314

[41] Ota KV Jamieson F Fisman DN: Prevalence of and risk factors for quinolone-resistant *Neisseria gonorrhoeae* infection in Ontario. *CMAJ.* 2009;180(3):287–90. 10.1503/cmaj.080222 19188626 PMC2630352

[42] de Vries HJ van der Helm JJ Schim van der Loeff MF: Multidrug-resistant Neisseria gonorrhoeae with reduced cefotaxime susceptibility is increasingly common in men who have sex with men Amsterdam, the Netherlands. *Euro Surveill.* 2009;14(37): pii: 19330. 10.2807/ese.14.37.19330-en 19761737

[43] Le Lin B Pastore R Liassine N: A new sexually transmitted infection (STI) in Geneva? Ciprofloxacin-resistant Neisseria gonorrhoeae, 2002–2005. *Swiss Med Wkly.* 2008;138(15–16):243–6. 18431700 10.4414/smw.2008.12029

[44] Koedijk FD van Veen MG de Neeling AJ: Increasing trend in gonococcal resistance to ciprofloxacin in The Netherlands, 2006–8. *Sex Transm Infect.* 2010;86(1):41–5. 10.1136/sti.2009.037135 19703843

[45] Unemo M Nicholas RA: Emergence of multidrug-resistant, extensively drug-resistant and untreatable gonorrhea. *Future Microbiol.* 2012;7(12):1401–22. 10.2217/fmb.12.117 23231489 PMC3629839

[46] Fairley CK Hocking JS Zhang L: Frequent Transmission of Gonorrhea in Men Who Have Sex with Men. *Emerging Infect Dis.* 2017;23(1):102–4. 10.3201/eid2301.161205 27983487 PMC5176237

[47] Mohammed H Mitchell H Sile B: Increase in Sexually Transmitted Infections among Men Who Have Sex with Men England, 2014. *Emerging Infect Dis.* 2016;22(1):88–91. 10.3201/eid2201.151331 26689861 PMC4696713

[48] Baker J Plankey M Josayma Y: The prevalence of rectal, urethral, and pharyngeal Neisseria gonorrheae and Chlamydia trachomatis among asymptomatic men who have sex with men in a prospective cohort in Washington, D.C. *AIDS Patient Care STDS.* 2009;23(8):585–8. 10.1089/apc.2008.0277 19591608 PMC2760271

[49] Kidd S Zaidi A Asbel L: Comparison of antimicrobial susceptibilities of pharyngeal, rectal, and urethral Neisseria gonorrhoeae isolates among men who have sex with men. *Antimicrob Agents Chemother.* 2015;59(5):2588–95. 10.1128/AAC.04476-14 25691638 PMC4394826

[50] STI department, Epidemiology and Surveillance Unit, Centre for Infectious Disease Control: Sexually transmitted infections including HIV, in the Netherlands in 2016.2017 Accessed November 29, 2017. Reference Source

[51] Turner KM Garnett GP Ghani AC: Investigating ethnic inequalities in the incidence of sexually transmitted infections: mathematical modelling study. *Sex Transm Infect.* 2004;80(5):379–85. 10.1136/sti.2003.007575 15459406 PMC1744908

[52] Beauté J Cowan S Hiltunen-Back E: Travel-associated gonorrhoea in four Nordic countries, 2008 to 2013. *Euro Surveill.* 2017;22(20): pii: 30537. 10.2807/1560-7917.ES.2017.22.20.30537 28537548 PMC5479976

[53] Abdullah AS Ebrahim SH Fielding R: Sexually transmitted infections in travelers: implications for prevention and control. *Clin Infect Dis.* 2004;39(4):533–8. 10.1086/422721 15356817

[54] Sundbeck M Agardh A Östergren PO: Travel abroad increases sexual health risk-taking among Swedish youth: a population-based study using a case-crossover strategy. *Glob Health Action.* 2017;10(1): 1330511. 10.1080/16549716.2017.1330511 28598729 PMC5496094

[55] Etkind P Ratelle S George H: International travel and sexually transmitted disease. *Emerging Infect Dis.* 2003;9(12):1654–6. 10.3201/eid0912.030210 14725311 PMC3034340

[56] Weir SS Feldblum PJ Roddy RE: Gonorrhea as a risk factor for HIV acquisition. *AIDS.* 1994;8(11):1605–8. 10.1097/00002030-199411000-00013 7848598

[57] Fingerhuth SM Bonhoeffer S Low N: Antibiotic-Resistant *Neisseria gonorrhoeae* Spread Faster with More Treatment, Not More Sexual Partners. *PLoS Pathog.* 2016;12(5):e1005611. 10.1371/journal.ppat.1005611 27196299 PMC4872991

[58] Mercer CH Prah P Field N: The health and well-being of men who have sex with men (MSM) in Britain: Evidence from the third National Survey of Sexual Attitudes and Lifestyles (Natsal-3). *BMC Public Health.* 2016;16:525. 10.1186/s12889-016-3149-z 27386950 PMC4936006

[59] Hassing RJ Alsma J Arcilla MS: International travel and acquisition of multidrug-resistant *Enterobacteriaceae*: a systematic review. *Euro Surveill.* 2015;20(47). 26625301 10.2807/1560-7917.ES.2015.20.47.30074

