# Supplementary material for: Epidemiological, behavioural, and clinical factors associated with antimicrobial-resistant gonorrhoea: a review
Source: F1000Res. 2018 Mar 27;7:400. [Version 1] doi: 10.12688/f1000research.13600.1 (PMC5871945; doi:10.12688/f1000research.13600.1)
Supplement: Supplementary file 1 [file f1000research-7-14773-s0000.tgz › b4c91d3d-158a-40d0-92f2-1d656952cf34.docx]

**Appendix 1. List of articles included in review, by year of publication**

| **Ref** | **First author, year of publication; location; sample size.**  **Study design and sampling method** | **Association with AMR-NG, as reported by authors**  **Yes No** | | | **Additional comments** |
| --- | --- | --- | --- | --- | --- |
| 22 | Hook EW III, 1989; Baltimore, USA; n=177.  Cross-sectional; consecutive sampling, prospectively enrolled patients seen for reasons other than test of cure at the Eastern STD Clinic of the Baltimore City Health Department | **TETRACYCLINE^a^**  Univariable analysis (comparison of proportions)  Men: >20 lifetime sexual partners (64%, 14/22 cases vs. 36%, 8/22 controls, p<0.05) (REF CAT 1-20 partners)  **PENICILLIN (CHROMOSOMAL)**  Univariable analysis  Men: >20 lifetime partners compared to cases with 1-20 partners (65%, 11/17 vs. 35%, 6/17, p<0.05), age ≥25 years (71%, 12/17 vs. 12%, 2/12, p<0.05) (REF CAT ≤19 years), reported past episodes of gonorrhoea (94%, 16/17 vs. 6%, 1/17, p<0.05) (REF CAT no previous episodes), >2 sexual partners in the past 30 days (29%, 5/17, vs. 24% 4/17, p<0.05) (REF CAT 0-1 partners), prostitute contact (23%, 13/17 vs. 77%, 4/17, p=0.06) (REF CAT no prostitute contact), parenteral drug use or sexual partners who were drug users (41%, 7/17 vs. 59%,10/17, p=0.07) (REF CAT no drug use) | | Univariable analysis  Men (n=125): antibiotic use in the past 2 weeks  Women (n=52): no association with any of the examined factors |  |
| 33 | Telzak EE,1989; New York, USA; n=547.  Cross-sectional; consecutive sampling, patients with a suspected gonorrhoea infection from the Central Harlem STD Clinic | **TETRACYCLINE**  Univariable analysis  Women (RR 2.0, 95% CI 1.03-4.06) (REF CAT men), patients who had been to a STI clinic (RR 1.9, CI 1.01-3.46) (REF CAT no previous visit to STI clinic), patients who took an oral antibiotic (RR 1.8, CI 0.97-3.44) (REF CAT no previous oral antibiotic use) | | Univariable analysis  Number of sexual partners, prostitute contact, presenting symptoms | Oral antibiotic use was reported as a positive finding of association but the confidence interval includes 1.0. |
| 38 | McLean CA, 2004; Kansas City, Missouri, USA; n=36.  Case-control nested within GISP; convenience sampling, 2 control patients were selected for each case patient for interviews and 4 control patients were selected for each case patient for medical record extraction | **Azithromycin (decreased susceptibility)**  Univariable analysis  Men: ≥1 sex partner(s) in the past month (OR 13.0, 95% CI 1.4-602, p<0.05) (REF CAT No new sex partner), met sex partner(s) on street A (OR 23, Cl 2.0-1102, p<0.01) (REF CAT not met sex partner(s) on street A), sex partner who received drugs or money for sex (OR 34.0, CI 2.3-1651, p<0.01) | | Univariable analysis  Consider Kansas City home, homeless at the time of interview, active-duty military, MSM, travel outside of Kansas City, nonprescription antibiotic use, antibiotic use, multiple sex partners, new sex partners, prior STI (syphilis, gonorrhoea, chlamydia, herpes), CSW exposure, incarceration, partner nonprescription drug use, partner working as CSW, partner travel outside Kansas and partner antibiotic use |  |
| 14 | Iverson CJ, 2004; Hawaii, USA; n=141.  Case-control; convenience sampling, 14 patients were diagnosed with ciprofloxacin resistant NG at an STI clinic and met the case-patient definition. 127 patients were included as controls | **Ciprofloxacin**  Univariable analysis  Recent foreign exposure in Asia (50%, 6/12 vs. 9%, 10/117, p=0.0008) (REF CAT no recent foreign exposure in Asia), foreign travel to Asia (33%, 4/12 vs. 7% 8/117, p=0.01) (REF CAT no recent foreign exposure to Asia), sex partner who recently travelled to Asia (17%, 2/12 vs. 2% 2/117, p=0.04) (REF CAT sex partner did not travel recently to Asia), antimicrobial use prior 30 days (21%, 3/14 vs. 3%, 4/124, p=0.02) (REF CAT no antimicrobial use prior to 30 days), black ethnicity (0%, 0/13 vs. 32%, 40/126, p=0.02) (REF CAT no black ethnicity)  Asian Pacific Islanders (91%, 10/11 vs 56%, 29/52, p=0.04) (REF CAT non-Asian Pacific Islander), originally from Asia (50%, 5/10 vs. 16%,7/43, p=0.04) (REF CAT not Asian) from USA other than Hawaii (0%, 0/11 vs. 42%, 18/43, p=0.01) (REF CAT US Hawaii), recent foreign exposure (30%, 3/10 vs. 4%, 2/52, p=0.03) (REF CAT no recent foreign exposure), recent foreign travel (30%, 3/10 vs. 4%, 2/52, p=0.03) (REF CAT no recent foreign travel) | | Univariable analysis  Year of diagnosis, median age, sex, sexual orientation, median number of sex partners, history of previous STI, history of previous gonorrhoea, self-recent foreign exposure, recent antimicrobial use, military occupation, military sex partner, CSW exposure, sex partner engaged in CSW | Recent foreign exposure is defined as travel within 30 days before diagnosis of gonorrhoea or a sex partner with foreign travel in the last 60 days.  We calculated the OR from the given data: foreign travel to Asia (OR 6.81, 95% CI 1.68-27.58, p=0.01), sex partner with a history of recent foreign travel (OR 11.5, CI 1.46-90.56, p=0.04) or recent antibiotic use (OR 0.12, CI 0.02-0.61, p=0.02), Asian Pacific Islander origin (OR 0.12, Cl 0.015-1.05, p=0.04), originally from Asia (OR 0.19, Cl 0.04-0.85, p=0.04), from USA other than Hawaii (OR Inf, CI NaN-Infinity, p=0.01), recent foreign exposure (OR 0.09, Cl 0.01-0.66, p=0.03) and recent foreign travel (OR 0.09, Cl 0.01-0.66, p=0.03) |
| 39 | Bauer HM, 2005; California, USA; n=952.  Cross-sectional nested within GISP; convenience sampling; patients with viable isolates tested for fluoroquinolone resistance at any of the 4 California GISP STI clinics | **FLUOROQUINOLONE**  Univariable analysis  Age 35-65 years (crude PR 2.0, 95% CI 1.1-3.9, p=0.04) (REF CAT 12-24 years), race/ethnicity not Asian or white (PR 0.5, CI 0.3-0.8, p=0.002) (REF CAT white), HIV positive cases (crude PR 2.3, CI 1.4-3.8, p=0.003) (REF CAT HIV negative), antibiotic use with in the prior month (OR 4.7, Cl 2.7-8.4 p<0.01) (REF CAT no antibiotic use), attendance at Long Beach or San Diego STD clinic (PR 2.7, Cl 1.4-5.4, p=0.004) (REF CAT San Francisco GISP site)  Multivariable analysis  Race/ethnicity not Asian or white (aOR 0.5, CI 0.3-1.0, p=0.05), recent antibiotic use (aOR 5.3, 95% CI 2.2-12.8, p=0.0002), MSM from San Diego STD clinic (aOR 4.6 Cl 1.0-20.3, p=0.05) (REF CAT non MSM at San Diego STD clinic), San Francisco clinic (aOR 0.2, Cl 0.04-0.6, p=0.01) (REF CAT non MSM at San Francisco clinic) | | Univariable analysis  MSM, CSW, travel outside USA or Hawaii, injection drug use, lack of symptoms, multiple partners  Multivariable analysis  GISP site | Measure of association is PR instead of OR |
| 40 | Farhi D, 2007; Paris, France; n=160.  Cross-sectional; consecutive sampling, all consecutive cases of gonorrhoea in a public venereology centre | **FLUOROQUINOLONE**  Univariable analysis  MSM (16.7% vs. 7.1%), HIV-infected patients (20.5% vs. 11.9%), ≥5 sex partners during the last year (24.4% vs. 17.1%)  Multivariable analysis  No associations were found  **Tetracycline**  Univariable analysis  Tetracycline resistance tended to be more frequent among heterosexuals (13.8% vs.5.9%)  Multivariable analysis  No associations were found | | None identified | Article does not show how the results of multiple regression were reached |
| 41 | Ota KV, 2009; Ontario, Canada; n=1,383.  Case-control; consecutive sampling, all quinolone-resistant isolates in Ontario were included as well as a random sample of quinolone-susceptible isolates. Controls were selected using a 1:1 case-to-control ratio | **FLUOROQUINOLONE**  Univariable analysis  Men (OR 3.1, 95% CI 2.3-4.1, p<0.001) ( REF CAT female), >30 years of age (OR 3.1, CI 2.4-3.8, p<0.001) (REF CAT <20 years), MSM (OR 1.4, CI 1.1-1.8, p=0.63) (REF CAT MSW), intermediate or resistant to penicillin (OR 7.4, Cl 3.9-14.0, p<0.001) (REF CAT penicillin susceptible NG), intermediate or resistant to tetracycline (OR 11.3, Cl 6.4-19.9, p<0.001) (REF CAT tetracycline susceptible NG), intermediate or resistant to erythromycin (OR 11.2, Cl 8.6-14.6, p<0.001) (REF CAT erythromycin susceptible NG) and multidrug resistant NG (OR 20.9, Cl 10.1-42.9, p<0.01) (REF CAT no multidrug resistant NG) | | None identified |  |
| 42 | de Vries HJC, 2009; Amsterdam, the Netherlands; n=1596.  Cross-sectional; consecutive sampling, all isolates with available MIC information collected from high-risk patients at a STI clinic in Amsterdam | **Cefotaxime (reduced susceptibility)**  Univariable analysis  >35 years (OR 1.8, 95% CI 1.2-2.7, p=0.004) (REF CAT <35), MSM (OR 3.2, CI 1.6-6.5, p<0.001) (REF CAT MSW), concurrent LGV infection (OR 2.7, CI 1.02-7.1, p=0.04) (REF CAT no LGV), new HIV diagnosis (OR 3.1, CI 1.3-7.3, p=0.023) (REF CAT HIV negative), known HIV status (OR 1.5 CI 1.0-2.4, p=0.023) (REF CAT HIV negative), positive TPHA serology (OR 1.7, Cl 1.1-2.7,p=0.01)  Multivariable analysis  MSM (OR 2.9, CI 1.4-5.8, p<0.001, adjusted for age) | | Univariable analysis  Concurrent infectious syphilis |  |
| 43 | Le Lin B, 2008; Geneva, Switzerland; n=91.  Case-control; convenience sampling, Geneva microbiology laboratories identified NG cases. Antimicrobial susceptibility testing was performed on a sample of reported cases | **Ciprofloxacin**  Univariable analysis  Sexual activity outside of Switzerland (OR 4.2, 95% CI 1.4-12.7, p=0.007)  Multivariable analysis  Sexual activity outside of Switzerland after adjusting for sexual orientation and nationality (OR 7.0, CI 1.99-24.6) | | Univariable and multivariable analysis  Age ≤40 years, Swiss nationality, MSM | Intermediate ciprofloxacin resistance was not considered in the analysis |
| 44 | Koedijk, FD, 2010; the Netherlands; n=1556.  Case-control nested within in GRAS; convenience sampling, isolates from the Dutch national STI surveillance network, which participates in GRAS | **Ciprofloxacin**  Univariable analysis  Men: ≥35 years (OR 1.6 95% CI 1.3-1.9) (REF CAT <35), MSM (OR 2.3, CI 1.8-2.9) (REF CAT MSW), HIV positive men (OR 1.5, CI 1.1-1.9), Dutch ethnicity (OR 1.6-1.3-2.0) (REF CAT non-Dutch ethnicity) and year of isolation 2008 (OR 1.6, Cl 1.1-2.3) (REF CAT 2006)  Women: >35 years (OR 6.0, CI 2.5-14.4) (REF CAT <35), CSW in the last 6 months (OR 25.2, CI 8.2-77.1) (REF CAT no exposure)  Multivariable analysis  Men: MSM (OR 2.0 CI 1.5-2.6), year of isolation 2008 (OR 1.6 Cl 1.1-2.4)  Women: >35 years (OR 8.2,CI 3.0-22.7), CSW (OR 25.0, CI 7.7-78.2) | | Univariable analysis  Women: year of consultation, Dutch ethnicity, positive HIV status  Multivariable analysis  Men: age ≥35 years, Dutch, positive HIV status  Women: year of isolation, Dutch ethnicity, HIV status |  |
| 23 | Ison CA, 2013; England and Wales; n=6176.  Case-control nested within GRASP; convenience sampling, isolates from GRASP and patients attending 26 genitourinary medicine clinics in England | **Cefixime (decreased susceptibility)**  Univariable analysis  20-24 years (OR 2.12, CI 1.39-3.2), 25-35 years (OR 4.34, 95% CI 2.92-6.47), 35-44 years (OR 5.67, CI 3.70-8.68), ≥45 years (OR 5.28, CI 3.33-8.33) (REF CAT 13-19), MSM (OR 7.77, CI 5.79-10.44) (REF CAT MSW), ≥2 sex partners (OR 1.52, CI 1.23-1.87) (REF CAT 0-1 partners), ≥6 partners (OR 2.89, CI 2.17-3.85) (REFCAT 0-1 partners), sex abroad (OR 1.36, CI 1.03-1.69), history of gonorrhoea (OR 1.55, CI 1.28-1.88), HIV positivity (OR 2.82, CI 2.26-3.52), year of isolation 2008 (OR 1.9, Cl 1.05-3.48), year of isolation 2009 (OR 7.68, Cl 4.51-13.06), year of isolation 2010 (OR 13.45, Cl 7.88-22.94), year of isolation 2011 (OR 7.87, Cl 4.62-13.42) (REF CAT 2007) were more likely to have decreased cefixime susceptibility  Women (OR 0.56, CI 0.34-0.90) (REF CAT MSW), people of black ethnic origin (OR 0.21, CI 0.15-0.30) (REF CAT white) and those with concurrent chlamydia infection (OR 0.45, CI 0.35-0.5) (REF CAT no infection) were negatively associated with decreased susceptibility to cefixime  Multivariable analysis  MSM (OR 5.47, CI 3.99-7.48, p<0.0001), year of isolation 2008 (OR 1.97, CI 1.02-3.81, p=0.04), year of isolation 2009 (OR 7.79, CI 4.42-13.7, p<0.0001), year of isolation 2010, (OR 13.08, CI 7.49-22.8, p<0.0001) and year of isolation 2011 (OR 6.72, CI 3.8-11.9, p<0.0001), women (OR 0.49, CL 0.29-0.81,P=0.006), black ethnic origin (OR 0.54, Cl 0.36-0.79, p=0.002), concurrent chlamydia infection (OR 0.71, CI 0.54-0.92, p=0.011) | | Univariable analysis  Symptoms present  Multivariable analysis  Age, number of sexual partners, sex abroad, previous gonorrhoea, positive HIV status |  |
| 24 | Trecker MA, 2014; Shanghai, China; n=384.  Cross-sectional; convenience sampling, Symptomatic male patients who tested positive for NG at the Shanghai Sexually Transmitted Infection and Skin Disease Hospital were included. A subsample of cases with complete AMR-NG data was used for the analysis | **Ceftriaxone (MIC >0.03mg/ml)**  Univariable analysis  Level of education (p=0.10), salary (p=0.23), male gender (p=0.06), use of drugs during sex (p=0.22) and number of partners (p=0.20)  Multivariable analysis  Men (OR 2.18, 95% CI 1.10-4.32, p=0.03) (REF CAT women)  **Ceftriaxone (MIC >0.125mg/ml)**  Univariable analysis  Use of over the counter drugs (p=0.28 ), age ≥38 years (p=0.29), male gender (p=0.27), drug use during sex (p=0.24)  Multivariable analysis  Age 46-83 years (OR 3.83, CI 1.33–11.01) (REF CAT 14-26 years), those who did not answer the question about over the counter antibiotic use (OR 0.25, Cl 0.07-0.88, p=0.03) (REF CAT did report over the counter antibiotic use)  **TETRACYCLINE**  Univariable analysis  Level of education (p=0.01), salary 1300-2000 Yuan (p=0.86), salary 22,000-35,000 Yuan (p=0.06), previous STI (p=0.12), male gender (p=0.11), alcohol use during sex (p=0.01)  Multivariable analysis  Men (OR 2.73, CI 1.06-4.23 p=0.03), alcohol use (OR 1.69, CI 1.08-2.64, p=0.02), middle-salary category (OR 0.34, Cl 0.14-0.18 p= 0.02) (REF CAT lower salary)  **PENCILLIN**  Univariable analysis  Level of education (p=0.09), reported previous bacterial STI (p=0.22), genital washing before and after sex (p=0.11), taking over the counter antibiotics (p=0.01)  Multivariable analysis  Participation in phase 2 of the study | | None identified | Univariable results are reported as p-values only |
| 25 | Cole MJ, 2014; Europe; n=5034.  Case-control study nested within GASP; convenience sampling, NG isolates from 21 countries participating in GASP | **Cefixime**  Univariable analysis  Year of isolation 2010 (OR 1.76 1.31-2.35), year of isolation 2011 (OR 1.53, Cl 1.14-2.05) (REF CAT 2009), male heterosexuals (OR 2.14 Cl 1.55-2.96), women (OR 1.55, Cl 1.08-2.23) (REF CAT MSM), no concurrent chlamydia (OR 2.08, Cl 1.26-3.44) (REF CAT concurrent chlamydia infection), previous gonorrhoea infection (OR 1.7, Cl 1.09-2.63), age ≥25 years (OR 1.3, Cl 1.02-1.65), anorectal site of infection (OR 0.63, Cl 0.42-0.95) (REF CAT genital), outpatient clinical services (OR 4.23, 2.79-6.42) (REF CAT STI service type)  Multivariable analysis  Heterosexual men (aOR 2.39, 95% CI, 1.58-3.61, p<0.001) (REF CAT MSM), women (aOR 2.75, CI 1.68-4.5, p<0.001) (REF CAT MSM), age >25 years (aOR 2.07, CI 1.36-3.13, p=0.001) (REF CAT <25), no concurrent chlamydial infection (aOR, 1.87, CI, 1.1-3.16, p=0.021) (REF CAT concurrent infection)  **Ciprofloxacin**  Univariable analysis  Male heterosexuals (OR 1.82, Cl 1.54-2.16) (REF CAT MSM), no current chlamydia infection (OR 2.56, Cl 2.04-3.21) (REF CAT no concurrent chlamydia infection), age >25 years (OR 1.49, Cl 1.32-1.68), no history of gonorrhoea (OR 1.35, Cl 1.08-1.7) (REF CAT history of gonorrhoea), anorectal site of infection (OR 0.7, Cl 0.58-0.84) (REF CAT genital), outpatient clinical service (OR 1.95, Cl 1.39-2.73) (REF CAT STI clinic), year of isolation 2010 (OR 0.66 Cl 0.57-0.76), year of isolation 2011 (OR 0.56, Cl 0.48-0.65) (REF CAT 2009)  Multivariable analysis  Heterosexual men (aOR 1.47, CI, 1.2-1.81, p<0.001), >25 years (aOR 1.7, CI, 1.39-2.08, p<0.001), no concurrent chlamydial infection (aOR 2.14, CI, 1.68-2.71, p<0.001), year of isolation 2010 (aOR 0.73, CI, 0.57-0.92, p=0.01), year of isolation 2011 (aOR 0.6, CI, 0.47-0.76, p<0.001)  **Azithromycin**  Univariable analysis  Male heterosexuals (OR 1.57, Cl 1.17-2.12) (REF CAT MSM), no current chlamydia infection (OR 2.32, Cl 1.36-3.94) (REF CAT concurrent chlamydia infection), previous history of gonorrhoea (OR 1.86, Cl 1.17-2.95) (REF CAT no previous history), year of isolation 2010 (OR 0.51, Cl 0.4-0.65), year of isolation 2011(OR 0.37, Cl 0.37-0.48) (REF CAT 2009)  Multivariable analysis  No concurrent chlamydial infection (aOR 2.18, CI 1.28-3.71, p=0.004), year of isolation 2010 (aOR 0.68, CI, 0.46-1 p=0.01), year of islation2011 (aOR 0.44, CI, 0.29-0.67, p<0.001) (REF CAT 2009) | | **Azithromycin**  Univariable analysis  Age ≥25, site of infection, clinical service type  **Cefixime**  Multivariable analysis  Previous gonorrhoea infection, year of isolation, site of infection, clinical service type  **Ciprofloxacin**  Multivariable analysis  Previous gonorrhoea infection, site of infection, clinical services type  **Azithromycin**  Multivariable analysis  Previous gonorrhoea infection, sexual orientation, age, site of infection, clinical service type |  |
| 26 | Plitt S, 2009; Alberta, Canada; n=200.  Cross-sectional; convenience sampling, laboratory surveillance data from 2 sentinel sites in Alberta | **Ciprofloxacin**  Univariable analysis  90% men, 77% white and 60% MSM. The median age was 29 years (interquartile range: 23-29 years) | | None identified | Ethnicity was extracted as a risk factor from text. We calculated the following OR: White ethnicity (OR 13.22, 95% Cl 6.47-26.99, p<0.001) |
| 27 | Wind CM, 2017; Amsterdam, the Netherlands; n=2318.  Cross-sectional; convenience sampling, consultations with a positive culture and MICs for azithromycin and ceftriaxone from a STI clinic in Amsterdam | | **Azithromycin (MIC >0.25 mg/L)**  Univariable analysis  MSM: year of isolation 2014 (OR 3.78, 95% CI 2.62-5.46, p<0.001), year of isolation 2013 (OR 1.56, Cl 1.04-2.35) (REF CAT 2012), HIV positivity (OR 0.70, CI 0.54-0.9, p<0.02) (REF CAT negative HIV), pharyngeal gonorrhoea (OR 1.46, Cl 1.02-2.18 p=0.02) (REF CAT urethral)  Heterosexual males and females: age ≥30 years (OR 3,83, Cl 1.05-13.99, p=0.02) (REF CAT ≤19), ≥10 sexual partners (REF CAT 0-1 sexual partners)  Multivariable analysis  MSM: HIV positive patients (OR 0.72, Cl 0.54-096, p=0.04), year of isolation 2014 (OR 3.83, Cl 2.64-5.55), year of isolation 2015 (OR 1.93, Cl 1.26-2.95) (REF CAT 2012)  Heterosexuals males and females: ≥10 partners (OR 6.74, CI 1.86-24.42, p<0.001) (REF CAT 0-1 partners)  **Ceftriaxone (MIC >0.032 mg/L)**  Univariable analysis  MSM: year of isolation 2013 (OR 2.56, Cl 1.63-4.02, p<0.001), year of isolation 2014 (OR 3.17, CI 2.04-4.91 p<0.001), year of isolation 2015(OR 1.87, Cl 1.13-3.08, p<0.001) (REF CAT 2012), pharyngeal infections (OR 2.56, CI 1.67-3.91, p<0.001) (REF CAT urethral gonorrhoea)  Heterosexual males and females: year of isolation 2014 or 2015 (OR 6.99, CI 2.21-22.11, p<0.001), females (OR 3.23, CI 1.55-6.74, p=0.007), ≥10 sex partners (OR 9.05, CI 3.01-27.21, p<0.001), pharyngeal gonorrhoea (OR 8.78 Cl 3.44-22.42, p<0.001), rectal gonorrhoea (5.71, 2.34-13.95, p<0.001) (REF CAT urethral), Surinamese origin (OR 0.33, Cl 0.11-0.98, p=0.002) (REF CAT Dutch), age ≤19 years (OR 0.14, Cl 0.03-0.65, p=0.02) (REF CAT ≥30)  Multivariable analysis  MSM: year of isolation 2014 (OR 3.0, Cl 1.92-4.66, p<0.001), year of isolation 2015 (OR 1.71, Cl 1.03-2.83) (REF CAT 2012), pharyngeal anatomical site (OR 2.52 Cl 1.64-3.89, p<0.001)  Heterosexual males and females: year of isolation 2014 (OR 5.44, Cl 1.71-17.23, p<0.001), year of isolation 2015 (OR 5.54, Cl 1.65-18.65, p<0.001), female (OR 3.14 Cl1.32-7.45, p=0.007), ≥10 partners (OR 6.16, CI 1.92-19.79, p=0.001) (REF CAT 0-1 partners) | **Azithromycin**  Univariable analysis  MSM: age, origin, number of sexual partners, previous syphilis, chlamydia infection, anatomical site  Heterosexual men and women:  year of diagnosis, sex, origin (Dutch or non-Dutch), anatomical site, HIV status, previous active syphilis, chlamydia, anatomical site  Multivariable analysis  Sex, age, number of anatomical sites  **Ceftriaxone**  Univariable anaylsis  MSM: Age, origin, number of sexual partners, HIV status, previous active syphilis, chlamydia, number of anatomical sites infected  Heterosexual men and women: Previous active syphilis, chlamydia, number of anatomic sites infected  Multivariable analysis  Age and origin | The multivariable analysis was not reported for all patient groups (e.g. MSM, MSW and women) together |
| 28 | Town K, 2017; England and Wales; n=8829.  Case-control nested within GRASP; consecutive sampling, consecutive gonorrhoea diagnoses that were cultured in 27 participating STI clinics | | **Ceftriaxone (MIC ≥0.015 mg/L)**  Univariable analysis  MSM: Asian ethnicity (crude OR 1.42, 95% CI 1.07-1.88) (REF CAT white), symptoms present (OR 0.76, Cl 0.65-0.89, p<0.05), previous gonorrhoea diagnosis (OR 1.34, 1.16-1.54) (REF CAT no previous gonorrhoea), year of isolation 2008 (OR 0.52, Cl 0.37-0.76, p<0.05), year of isolation 2009 (OR 1.86, Cl 1.39-2.49, p<0.05), year of isolation 2010 (OR 1.98, Cl 1.48 2.63, p<0.05), year of isolation 2012 (OR 0.75, Cl 0.57-0.99, p<0.05) (REF CAT 2007)  Heterosexual men and women: >35 years of age, (OR 4.31, CI 3.34-5.55), 25-35 years (OR 1.07 Cl 0.90-1.28, p<0.05) (REF CAT <25), ≥6 sexual partners (OR 1.58, Cl 1.01-2.44) (REF CAT 0-1 partners), sex abroad in the last 3 months (OR 2.23, CI 1.71-2.91) (REF CAT no sex abroad in the last 3 months), black Caribbean (OR 0.29, CI 0.20-0.41), African ethnicity (OR 0.66, 0.43-0.99) (REF CAT white), concurrent chlamydial infection (OR 0.25, CI 0.19-0.34) (REF CAT no concurrent infection), symptomatic cases (OR 0.25, Cl 0.19-0.34, p<0.05) (REF CAT no symptom), previous gonorrhoea infection (OR 0.75, Cl 0.62-0.92, p<0.05), positive HIV status (OR 3.56, Cl 1.70-7.46, p<0.05), year of isolation 2009 (OR 2.09, Cl 1.44-3.05, p<0.05), year of isolation 2012 (OR 1.62, Cl 1.07-2.44, p<0.05), year of isolation 2013 (OR 2.88, 1.97-4.20, p<0.05) (REF CAT 2007), isolation site rectal (OR 3.18, Cl 1.85-5.48, p<0.05), isolation site throat (OR 5.26, Cl 3.01-9.17, p<0.05) (REF CAT genital), women (OR 0.57, CI 0.46-0.71, p<0.05)  Multivariable analysis  MSM: symptomatic gonococcal infection (aOR 0.77, CI 0.66-0.90), year of isolation 2008 (aOR 0.55, CI 0.38-0.80) (REF CAT 2007), year of isolation 2009 (aOR 1.88, CI-1.40-2.52), year of isolation 2010 (aOR 1.98, CI 1.48-2.64) (REF CAT 2007)  Heterosexual men and women: age 25-34 years (aOR 1.56, CI 1.34-1.82), ≥35 years (aOR 1.96, CI 1.66-2.31), year of isolation 2009 (aOR 1.89, CI 1.50-2.39), year of isolation 2010 (aOR 1.85, CI 1.46-2.39), year of isolation 2011 (aOR 1.42, CI 1.12-1.81), rectal infection (aOR 1.69, CI 1.46-1.96), throat infection (aOR 1.84, CI 1.44-2.34) (REF CAT genital isolation site), women (aOR 0.42, CI 0.34-0.52) (REF CAT men), black Caribbeans (aOR 0.36, CI 0.28-0.47), black African or black other ethnicity (aOR 0.32, CI 0.20-0.51) (REF CAT white), concurrent chlamydial infection (aOR 0.58, CI 0.50-0.68), year of isolation 2008 (aOR 0.60, CI 0.45-0.80) (REF CAT 2007) | Univariable analysis  Age, number of sexual partners, sex abroad, concurrent STI, HIV status, isolation site  Multivariable analysis  Ethnicity, previous gonorrhoea infection, number of sexual partners, prior to diagnosis, sex abroad, presence of symptoms, HIV status | The multivariable analysis was not reported for all patient groups (e.g. MSM, MSW and women) together |
| 29 | Farhi D, 2009; Paris, France; n=110.  Cross-sectional; consecutive sampling, patients diagnosed with NG in 9 STI clinics | | **FLUOROQUINOLONE**  No risk factors were identified | Gender, age, recent sexual partner, past history of STI, susceptibility to penicillin or tetracycline | Not clear if results are univariable or multivariable |
| 30 | Goldstein E, 2012; USA; n=not reported.  Cross-sectional nested within GISP; convenience sampling, Isolates with resistance from GISP | | **Ciprofloxacin, tetracycline and penicillin**  Multivariable analysis  MSM: triple resistance and recent travel (OR 0.72, 95% CI 0.52-0.99) (REF CAT no recent travel history)  MSW: triple resistance and recent travel (OR 3.22, CI 2.24-4.62), mono-resistance and recent travel (OR 1.72, CI 0.98-3.03) | None identified |  |
| 31 | Klausner JD; 1999; Philippines; n=1499.  Cross-sectional; convenience sampling, women attending 3 social hygiene clinics in metropolitan Manila and 1 social hygiene clinic | | **Ciprofloxacin**  Univariable analysis  Marital status, living alone, duration of sex work, and clinic site were identified as risk factors. AMR isolates were found in 10 (11.5%) of 87 CSWs reporting self-prescribed antimicrobial use vs. 44 (3.4%) of 1295 reporting no antimicrobial use (p<.001)  Multivariable analysis  High level AMR was associated with CSW from metropolitan Manila (OR 4.9, 95% CI, 3.2-7.4) (REF CAT Cebu city), working as a CSW <12 months (OR 2.4, Cl 1.5-3.7), self-prescribed antibiotic use (OR 1.3, Cl 0.7-2.5) (REF CAT no antibiotic use) | None identified | PR or OR values are not reported for marital status, living alone, duration of sex work or clinical site  No clear comparison group for “working as CSW < 12 months” |
| 32 | Lahra MM, 2017; Australia; n=5411.  Cross-sectional nested within AGSP; convenience sampling, Clinical isolates tested for antimicrobial susceptibility and submitted to AGSP | | **PENICILLIN (PPNG)**  PPNG rates remain low in the remote regions of Western Australia. All PPNG positive NG from remote regions were determined to be in non-indigenous residents or residents in the major regional centres (2/120, 1.6%, from the Pilbara and 0/341, 0% from the Kimberley, and lower rates in Midwest 4.7% and Goldfields 0%). There was no PPNG detected in the remote Indigenous community (personal communication from Dr David Speers, PathWest) | None identified | Ethnicity and geographical variation were extracted as risk factors from the text |
| 34 | Speers DJ, 2014; Australia; n=1235.  Cross-sectional; convenience sampling, specimens from remote regions of Western Australia | | **PENICILLIN**  In remote regions of Western Australia where gonorrhoea is highly endemic, 5% of isolates were PPNG. This contrasts with rates of up to 20% observed in the more densely populated metropolitan and rural regions | None identified | Geographical difference was extracted as a risk factor from text |
| 35 | Fuertes de Vega I, 2017; Barcelona, Spain; n=101.  Cross-sectional; convenience sampling, patients recruited from a tertiary care hospital in Barcelona | | **Ciprofloxacin**  Multivariable analysis  MSM (OR 0.30, p=0.03) and older patients (OR 1.46, p=0.03)  **Cefotaxime**  Multivariable analysis  HIV positive patients (OR 0.12, p=0.05), higher number of sexual partners in previous 3 months (p=0.008) (REF CAT 0-1 partners), older patients (OR 2.13, p=0.05), those who reported foreign sex (OR 26.9, p=0.02), co-infection with other STI (OR 29.9, p=0.02), older patients (OR 1.47, p=0.04), previous gonorrhoea in the last 12 months (OR 4.92, p=0.04) | None identified | The cut-off for older age is not reported. |
| 36 | Zhu BY, 2014; Nanning, China, n=923.  Cross-sectional; consecutive sampling, Isolates were collected by clinicians from patients with uncomplicated gonorrhoea attending an STI clinic | | **Ceftriaxone**  Univariable analysis  Age 16-25 years, (OR 2.32, 95% Cl 1.126-4.814) (REF CAT ≥50), isolation year 2008 (OR 3.656, Cl 1.545-8.650, p=0.03), isolation year 2009 (OR 11.532, Cl 4.876-27.277, p<0.001), isolation year 2010 (OR 4.301, Cl 1.575-11.743, p=0.04), isolation year 2012 (OR 5.919, Cl 2.188-16.013, p<0.001) (REF CAT 2007)  Multivariable analysis  Age 16-25 years (OR 2.589 CI 1.173-5.703, p=0.019) (REF CAT ≥50), year of isolation 2008 (OR 3.679, CI 1.450-9.335, p=0.006), year of isolation 2009 (OR 12.259 Cl 4.889-30.743, p<0.001), year of isolation 2010 (OR 4.768, CI 1.637-13.886; p=0.004), year of isolation 2012 (OR 7.173 CI, 2.390-21.524, p<0.001) (REF CAT 2007) | Univariable analysis  Age >25 years, sex, marital status, history of gonorrhoea, isolation year 2011 |  |
| 37 | Cole MJ, 2017; Europe; n=2134.  Cross-sectional nested within EURO-GASP, convenience sampling, isolates were collected from 24 participating countries in Euro-GASP | | **Cefixime**  Heterosexual men (OR 0.3, 95% CI 0.08-0.8, p<0.01), MSM (OR 0.1, CI 0.03-0.4, p<0.01 ) (REF CAT women) in 2015  **Azithromycin**  Previous gonorrhoea diagnosis (OR 2.1, CI 1.2–3.5, p<0.01)  **Ciprofloxacin**  Heterosexual men (OR 1.9, Cl 1.5-2.4, p <0.01) (REF CAT MSM) | None identified | Not clear if results are univariable or multivariable |

a. Articles reporting in terms of antibiotic class are written in block letters and specific antibiotics with in class are written in small letters. Cut-offs defining antimicrobial resistance are those reported by the authors of each study

Abbreviations: AGSP, Australian Gonococcal Surveillance Programme; AMR, Antimicrobial resistance; aOR, Adjusted odds ratio; CI, Confidence interval; CSW, Commercial sex worker; GASP, Gonococcal Antimicrobial Surveillance Programme; GISP, Gonococcal Isolate Surveillance Project; GRAS, Gonococcal Resistance to Antimicrobials Surveillance; GRASP, Gonococcal Resistance to Antimicrobials Surveillance Programme; HIV, Human immunodeficiency virus; LGV, Lymphogranuloma venereum; MIC, Minimum inhibitory concentration; MSM, Men who have sex with men; MSW, Men who have sex with women; NG, *Neisseria gonorrhoeae*; OR, Odds ratio; PPNG, Penicillinase-producing *Neisseria gonorrhoeae*; PR, Prevalence ratio; REF CAT, Reference category; RR, Relative risk; STD, Sexually transmitted disease; STI, Sexually transmitted infection; TPHA, *Treponema pallidum* haemagglutination; UK, United Kingdom; USA, United States of America
