# Supplementary material for: Epidemiological, behavioural, and clinical factors associated with antimicrobial-resistant gonorrhoea: a review
Source: F1000Res. 2018 Mar 27;7:400. [Version 1] doi: 10.12688/f1000research.13600.1 (PMC5871945; doi:10.12688/f1000research.13600.1)
Supplement: Supplementary file 2 [file f1000research-7-14773-s0001.tgz › a8bf35d8-5a80-4556-bffb-0a8491ed72a2.docx]

| **Appendix 2. Risk factors examined and associations with AMR-NG, as reported by authors of included studies** | | | | | | | |
| --- | --- | --- | --- | --- | --- | --- | --- |
| **Risk factors^a^** | **Number of articles that mention the characteristic** | | | | **Number of articles that did not mention the characteristic** | **Antibiotic class^d^** | |
|  | **Total number of articles that mention the characteristic^b^** | | **Number of articles that report an association with AMR-NG^c^** | **Number of articles that report no association with AMR-NG** |  | **Associated with any risk factor** | **Not associated with any risk factor** |
| **Epidemiological factors** | | | | | | | |
| Age (older or younger) | | 15 | 12  [22,23,24,25,27,28,35,36,39,41,42,44] | 9  [14,23,25,27,28,29,36,43,44] | 9 | TETRACYCLINE, PENICILLINS, FLUOROQUINOLONES, MACROLIDES, CEPHALOSPORINS | FLUOROQUINOLONES, CEPHALOSPORINS, MACROLIDES |
| Sex (men or women) | | 11 | 8  [23,24,25,27,28,33,35,41] | 4  [14,27,29,36] | 13 | TETRACYCLINES, FLUOROQUINOLONES, CEPHALOSPORINS | FLUOROQUINOLONES, CEPHALOSPORINS |
| Racial or ethnic group | | 8 | 7  [14,23,26,28,32,39,44] | 3  [27,28,44] | 16 | PENICILLINS, FLUOROQUINOLONES, CEPHALOSPORINS | FLUOROQUINOLONES, MACROLIDES, CEPHALOSPORINS |
| Sexual orientation (MSM or heterosexual men) | | 12 | 9  [23,25,35,37,39,40,41,42,44] | 4  [14,38,39,43] | 12 | FLUOROQUINOLONES, CEPHALOSPORINS, MACROLIDES | FLUOROQUINOLONES, MACROLIDES |
| Socioeconomic position | | 1 | 1  [24] | 0 | 23 | TETRACYCLINES, CEPHALOSPORINS |  |
| **Behavioural factors** | | | | | | | |
| Multiple sex partners and new partners | | 12 | 8  [22,23,24,27,28,35,38,40] | 5  [14,29,33,38,39] | 12 | PENICILLINS, FLUOROQUINOLONES, CEPHALOSPORINS, MACROLIDES | TETRACYCLINES, MACROLIDES, FLUOROQUINOLONES, CEPHALOSPORINS |
| Sex with partners abroad or travel history | | 10 | 6  [14,23,28,30,35,43] | 7  [14,23,27,28,29,38,39] | 14 | FLUOROQUINOLONES, CEPHALOSPORINS, TRIPLE ANTIBIOTICS (CIPROFLOXACIN, TETRACYCLINE AND PENICILLIN) | FLUOROQUINOLONES, MACROLIDES, CEPHALOSPORINS |
| Exchanging sex for money or prostitute contact | | 7 | 4 [22,31,38,44] | 4  [14,33,38,39] | 17 | PENICILLINS, FLUOROQUINOLONES, MACROLIDES | TETRACYCLINES, FLUOROQUINOLONES, MACROLIDES |
| Alcohol and drug use | | 4 | 3  [22,24,38] | 1  [39] | 20 | PENICILLINS, FLUOROQUINOLONES, TETRACYCLINES, MACROLIDES | FLUOROQUINOLONES |
| Life time sex partners | | 1 | 1  [22] | 0 | 23 | TETRACYCLINES |  |
| Type of relationship (casual or not) | | 2 | 1  [31] | 1  [36] | 22 | FLUOROQUINOLONES | CEPHALOSPORINS |
| **Clinical factors** | | | | | | | |
| Anatomical site of infection | | 3 | 3  [25,27,28] | 2  [25,27] | 21 | FLUOROQUINOLONES, CEPHALOSPORINS, MACROLIDES | MACROLIDES, CEPHALOSPORINS, FLUOROQUINOLONES |
|  |  |  |  |  |  |  |  |
| Co-infection with HIV or STIs | | 9 | 9  [23,25,27,28,35,39,40,42,44] | 5  [23,27,28,42,44] | 15 | FLUOROQUINOLONES, CEPHALOSPORINS, MACROLIDES | FLUOROQUINOLONES, CEPHALOSPORINS, MACROLIDES |
| Recent antibiotic use | | 6 | 4  [14,31,33,39] | 3  [14,22,38] | 18 | TETRACYCLINES, FLUOROQUINOLONES | TETRACYCLINES, PENICILLINS, FLUOROQUINOLONES, MACROLIDES |
| Number of gonorrhoea episodes/prior history of gonorrhoea | | 8 | 6  [22,23,25,28,35,37] | 5  [14,23,25,28,36] | 16 | PENICILLINS, FLUOROQUINOLONES, CEPHALOSPORINS, MACROLIDES | FLUOROQUINOLONES, CEPHALOSPORINS, MACROLIDES |
| Year of isolation | | 7 | 6  [23,25,27,28,36,44] | 5  [14,25,27,36,44] | 17 | FLUOROQUINOLONES, CEPHALOSPORINS, MACROLIDES | CEPHALOSPORINS, MACROLIDES, FLUOROQUINOLONES |
| History of STIs | | 5 | 1  [24] | 4  [14,27,28,29,38] | 19 | TETRACYCLINES, PENCILLINS | FLUOROQUINOLONES, CEPHALOSPORINS, MACROLIDES |
| More than one infection site | | 0 | 0 | 0 | 24 |  |  |

1. Risk factor identified by the original authors
2. Same risk factor could be associated or not associated with different antimicrobials in the same article. Therefore, the total number of articles that mention the risk factor is not the exact summation of number of articles that report association and no association
3. The numbers in square brackets correspond to the numbered references in the main text
4. Antimicrobial class in upper case, specific antimicrobials examined within in each class in lower case: TETRACYCLINES (tetracycline); PENICILLINS (penicillin); FLUOROQUINOLONES (ciprofloxacin); CEPHALOSPORINS (ceftriaxone, cefixime, cefotaxime); MACROLIDES (azithromycin)

**Abbreviations:** AMR-NG, Antimicrobial resistant *Neisseria gonorrhoeae*; STI, Sexually transmitted infection; MSM, Men who have sex with men
