# Supplementary material for: Epidemiological, behavioural, and clinical factors associated with antimicrobial-resistant gonorrhoea: a review
Source: F1000Res. 2018 Mar 27;7:400. [Version 1] doi: 10.12688/f1000research.13600.1 (PMC5871945; doi:10.12688/f1000research.13600.1)
Supplement: Supplementary file 3 [file f1000research-7-14773-s0002.tgz › fca8bdc0-6a33-4373-b032-1cf9d54e8d1b.docx]

**Appendix 3. Comparison between risk factors for gonorrhoea and factors associated with AMR-NG in this review**

| AMR-NG association^a^ | Gonorrhoea risk factors^b^ | | | | | | | | | | |
| --- | --- | --- | --- | --- | --- | --- | --- | --- | --- | --- | --- |
|  | Age (younger age associated) | Sex (male) | Sex partners (MSM) | Racial or ethnic group  (Black minority) | Low socioeconomic  position | New or multiple sex partners (association) | Sex with partners abroad | Exchange sex for money | Alcohol and drug use | Co-infection (CT more likely) | Prior history of gonorrhoea |
| Age (older associated) |  |  |  |  |  |  |  |  |  |  |  |
| Sex (male) |  |  |  |  |  |  |  |  |  |  |  |
| Sex partners (MSM) |  |  |  |  |  |  |  |  |  |  |  |
| Race/ethnicity (white) |  |  |  |  |  |  |  |  |  |  |  |
| Low SEP |  |  |  |  |  |  |  |  |  |  |  |
| Multiple sex partners (no association) |  |  |  |  |  |  |  |  |  |  |  |
| Sex partners abroad |  |  |  |  |  |  |  |  |  |  |  |
| Exchanging sex for money |  |  |  |  |  |  |  |  |  |  |  |
| Alcohol and drug use |  |  |  |  |  |  |  |  |  |  |  |
| Co-infection (CT less likely) |  |  |  |  |  |  |  |  |  |  |  |
| Prior history of gonorrhoea |  |  |  |  |  |  |  |  |  |  |  |

1. Risk factors examined in our review. Factors not included in the table but examined in the review were: number of lifetime sex partners, type of partnership, number of anatomical sites infected, year of isolation.
2. Risk factors identified from literature, but not reviewed systemically.

Red cells indicate inconsistency between risk factors for gonorrhoea and AMR-NG (direction of association shown in brackets)

Green cells indicate consistency between risk factor for gonorrhoea and AMR-NG

Text in yellow are additional risk factors, which are not common to both.

Abbreviations: AMR-NG, Antimicrobial resistant *Neisseria gonorrhoeae;* MSM, Men who have sex with men; SEP, socioeconomic position
